# Melatonin Alleviates Hyperglycemia-Induced Cardiomyocyte Apoptosis via Regulation of Long Non-Coding RNA H19/miR-29c/MAPK Axis in Diabetic Cardiomyopathy

**DOI:** 10.3390/ph15070821

**Published:** 2022-07-02

**Authors:** Haitao Tang, Hongli Zhong, Wanqing Liu, Yi Wang, Yuan Wang, Liuqing Wang, Songtao Tang, Huaqing Zhu

**Affiliations:** 1 Department of Immunology, School of Basic Medical Sciences, Anhui Medical University, Hefei 230032, China; tht199536@163.com; 2 Laboratory of Molecular Biology, Department of Biochemistry, Anhui Medical University, Hefei 230032, China; wanqing023@163.com (W.L.); wangyi@ahmu.edu.cn (Y.W.); aydesm-1@163.com (Y.W.); 3 General Department of Hyperbaric Oxygen, Hefei Hospital Affiliated to Anhui Medical University, Hefei 230000, China; zzhj7412@163.com; 4 Department of Clinical Laboratory, The Third Clinical School of Hefei of Anhui Medical University, Hefei 230022, China; 5 Department of Endocrinology, The First Affiliated Hospital of Anhui Medical University, Hefei 230022, China

**Keywords:** melatonin, diabetic cardiomyopathy, apoptosis, non-coding RNA, MAPK

## Abstract

Recent studies revealed that non-coding RNAs (ncRNAs) play a crucial role in pathophysiological processes involved in diabetic cardiomyopathy (DCM) that contribute to heart failure. The present study was designed to further investigate the anti-apoptotic effect of melatonin on cardiomyocytes in diabetic conditions, and to elucidate the potential mechanisms associated with ncRNAs. In animal models, we induced diabetes in SD rats by single intraperitoneal injection of streptozotocin (STZ) solution (55 mg/kg) at 18:00 in the evening, after a week of adaptive feeding. Our results indicate that melatonin notably alleviated cardiac dysfunction and cardiomyocyte apoptosis. In the pathological situation, lncRNA H19 level increased, along with a concomitant decrease in miR-29c level. Meanwhile, melatonin significantly downregulated lncRNA H19 and upregulated miR-29c levels. In our in vitro experiments, we treated H9c2 cells with high-concentration glucose medium (33 mM) to simulate the state of diabetes. It was verified that positive modulation of miR-29c and inhibition of lncRNA H19, as well as mitogen-activated protein kinase (MAPK) pathways, distinctly attenuated apoptosis in high-glucose-treated H9c2 cells. A luciferase activity assay was conducted to evaluate the potential target sites of miR-29c on lncRNA H19 and MAPK13. LncRNA H19 silencing significantly downregulated the expression of miR-29c target gene MAPK13 by inducing miR-29c expression. Most importantly, our results show that melatonin alleviated apoptosis by inhibiting lncRNA H19/MAPK and increasing miR-29c level. Our results elucidate a novel protective mechanism of melatonin on diabetic cardiomyocyte apoptosis, which involved the regulation of lncRNA H19/miR-29c and MAPK pathways, providing a promising strategy for preventing DCM in diabetic patients.

## 1. Introduction

Diabetes mellitus (DM) has become one of the most prevalent metabolic disorders in recent years. Cardiovascular complications are the leading cause of mortality and morbidity in diabetes. Diabetic cardiomyopathy (DCM), which develops in the absence of overt myocardial ischemia or hypertension, can trigger heart failure, and seriously endangers the lives of patients [1,2,3]. It has been reported that DCM can cause cardiac systolic and diastolic dysfunction, and various metabolic disorders, which leads to the stress response of cardiomyocytes and, ultimately, death [4,5]. The precise pathogenesis of DCM is still unclear, although cardiomyocyte apoptosis is considered to be one of the major mechanisms. There is strong evidence that alleviation of cardiomyocyte apoptosis is effective for the prevention and treatment of DCM [6,7,8,9,10].

It has been proven, by sequencing experiments, that more than 90% of the genome is transcribed into non-coding RNAs. Long non-coding RNAs (lncRNAs) and microRNAs (miRNAs) are two different kinds of non-coding RNAs. Interestingly, lncRNAs can act as sponges, binding to miRNAs, and thus, affect the function of target miRNAs; in contrast, miRNAs bind to the 3′-UTR of target genes to regulate their expression at the post-transcriptional level. Non-coding RNAs (ncRNAs) are involved in the control of multiple cellular activities, such as proliferation, differentiation, and apoptosis [11,12,13]. Numerous studies have suggested that ncRNAs play a key role in regulating pathophysiological processes involved in DCM, and the aberrant expression of lncRNAs/miRNAs may have repercussions on cardiomyocyte activities [14,15]. For the past few years, it has been extensively reported that lncRNA H19 and miRNA-29c both play a vital role in the development and progression of cardiovascular diseases. For example, lncRNA H19 is upregulated during the progression of atherosclerosis via the regulation of the MAPK and NF-κB signaling pathways [16,17]. Meanwhile, miRNA-29c decreased myocardial ischemia-reperfusion injury through the inhibition of immoderate autophagy [18]. MicroRNA-29c also inhibited migration and angiogenesis of human endothelial cells by depressing insulin-like growth factor 1 [19]. However, the related mechanisms of lncRNA H19/miR-29c in DCM need to be further investigated.

Melatonin (MLT, N-acetyl-5-methoxytryptamine), a pleiotropic molecule, is primarily secreted by the pineal gland, which is known for its antioxidant, anti-inflammatory, and anti-apoptotic effects [8,20,21,22]. Because of its function in scavenging free radicals directly, melatonin plays a critical role in alleviating many chronic diseases [23,24]. Evidence has been presented for the role of melatonin in the pathological process of cardiovascular disease. Yu et al. found that melatonin attenuated DCM and reduced myocardial ischemia-reperfusion injury by improving mitochondrial quality control via SIRT6 [25]. Ding et al. reported that melatonin participated in preventing Drp1-mediated mitochondrial fission by regulating the SIRT1-PGC1α pathway [26]. In our previous research, melatonin not only prevented diabetic cardiomyocyte apoptosis by inhibiting endoplasmic reticulum stress, but also ameliorated diabetic arterial endothelial permeability by regulating MAPK signaling pathway activation [27,28]. Intriguingly, previous studies have demonstrated the intimate relationship between cardiomyocyte apoptosis and MAPK signal pathways. Coincidentally, it has been reported in the literature that inhibiting MAPK signaling could reduce cardiomyocyte apoptosis induced by high glucose levels. For example, it was confirmed that angiotensin-(1–7) protected cardiomyocytes against high-glucose-induced injury and apoptosis by inhibiting ROS-activated leptin–p38 MAPK and ERK1/2 pathways [29]. Zhao et al. found that exogenous hydrogen sulfide ameliorated hyperglycemia-induced myocardial injury, inflammation, and apoptosis via the CIRP-MAPK signaling pathway [30]. On the basis of the above evidence, we hypothesized that melatonin could exert an ameliorative effect on cardiomyocyte apoptosis in DCM by blocking MAPK signal pathways.

Since ncRNAs show potential as biomarkers in DCM, the interaction between ncRNAs and MAPK signal pathways in cardiomyocytes remains an open question. Moreover, it has not yet been fully explored as to whether the beneficial effect of melatonin on cardiomyocyte apoptosis in DCM is mediated by the regulation of ncRNA networks. Thus, based on the above hypothesis and questions, we developed a DCM model in vivo and in vitro to broaden our understanding of the role of lncRNA H19/miRNA-29c/MAPK in the function of melatonin in alleviating cardiomyocyte apoptosis.

## 2. Results

### 2.1. Diabetes Influenced Body Weight and Blood Glucose of Rats, and Melatonin Improved Myocardial Fiber Disorder, Collagen Accumulation, and Myocardial Dysfunction in Diabetes

**Figure 1:**
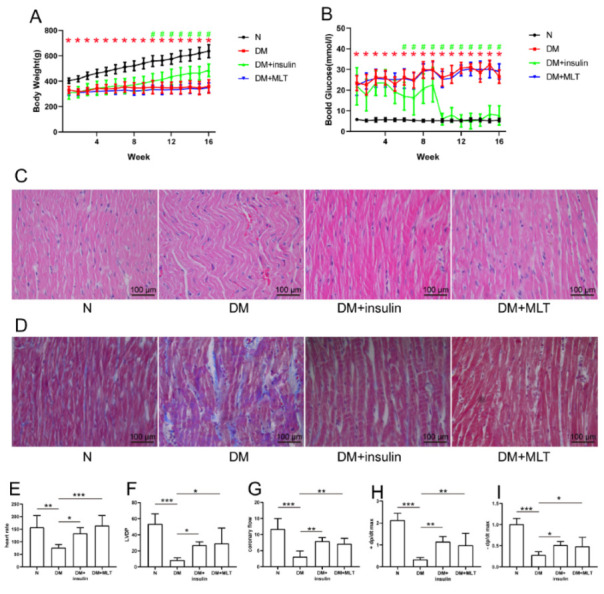
The body weight (**A**) and blood glucose (**B**) parameters of rats in different experimental groups (*n* = 12 per group). The parameters were measured by electronic scale and glucometer once a week. (**C**) H&E staining showed that melatonin improved the cardiac fiber disorder in diabetes-induced rats. (**D**) Masson’s trichrome staining showed that melatonin alleviated cardiac collagen accumulation caused by diabetes**.** Melatonin relieved myocardial dysfunction in diabetic rats. The Langendorff perfusion system was used to record myocardial dysfunction in rats (*n* = 6 per group). (**E**) Heart rate; (**F**) LVDP; (**G**) coronary flow; (**H**) +dp/dt max; (**I**) −dp/dt max. The data are presented as means ± standard deviation (**A**,**B**,**E**–**I**) and representative images (**C**,**D**). All experiments were performed in triplicate. * *p* < 0.05 vs. normal group; # *p* < 0.05 vs. DM group (**A**,**B**). * *p* < 0.05, ** *p* < 0.01, *** *p* < 0.001 (**E**–**I**). Statistical analyses were performed using one-way ANOVA with Tukey’s multiple comparisons test.

During the experiments, we measured the body weight and blood glucose of the rats once a week; these measurements were taken after insulin and melatonin treatment for 4 h, using an electronic scale and blood glucose meter, respectively. The body weights of diabetic rats increased much slower than in the normal group (Figure 1A), and blood glucose levels were consistently high (Figure 1B). Insulin treatment facilitated body weight gain and decreased blood glucose levels in diabetic rats. After 10 weeks, we treated rats with insulin twice a day in the DM + insulin group. During the experiments, we found that, with the development of diabetes, insulin treatment once a day was not enough to decrease the blood glucose levels of diabetic rats significantly; therefore, we administered insulin twice a day, and found that the blood glucose could be reduced to near normal levels. However, melatonin administration did not have these effects (Figure 1A,B). H&E and Masson’s trichrome staining showed that long-term diabetes significantly impaired myocardial fibers (Figure 1C) and aggravated collagen accumulation (Figure 1D). Interestingly, insulin and melatonin treatment could significantly improve myocardial tissue fibrosis and collagen accumulation caused by hyperglycemia, which illustrated that the treatment with insulin and melatonin cured diabetes-induced pathological injury (Figure 1C,D). Additionally, heart rate, left ventricular development pressure (LVDP), coronary flow, maximal rate of increase in left ventricular pressure (+dp/dt max), and maximal rate of the decrease in left ventricular pressure (−dp/dt max), were distinctly decreased in the DM group compared with the normal group (Figure 1E–I). These results indicate that long-term hyperglycemia contributed to myocardial dysfunction. Nevertheless, melatonin treatment improved cardiac function remarkably.

### 2.2. Melatonin Alleviated Cardiomyocyte Apoptosis Caused by Hyperglycemia

**Figure 2:**
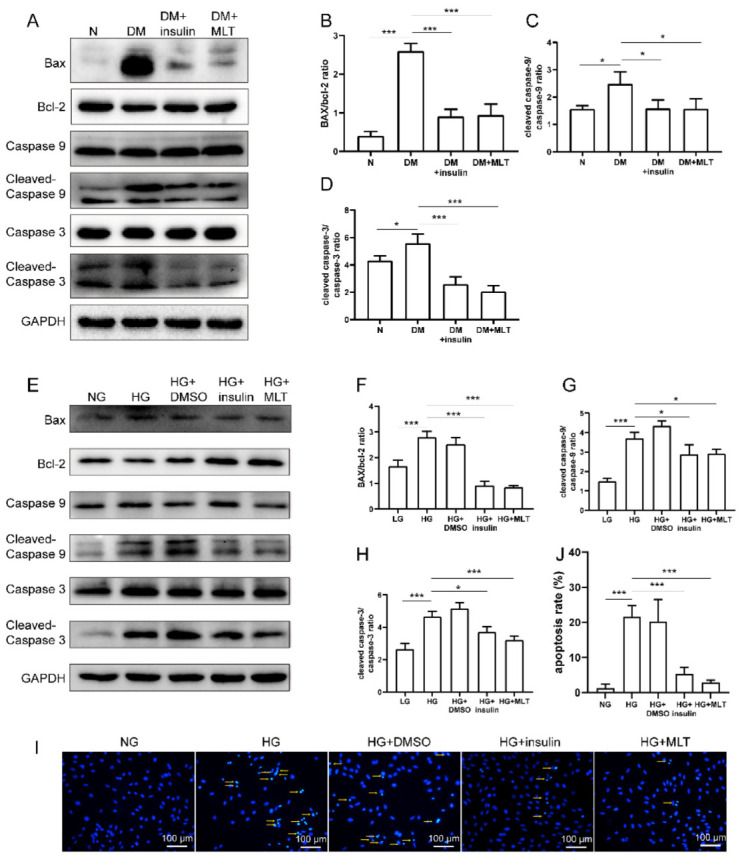
Melatonin alleviated apoptosis of cardiomyocyte in vitro and vivo. (**A**–**D**) Western blotting was used to detect the expression levels of cleaved caspase-3, cleaved caspase-9, and Bax/Bcl-2 in the myocardium of different groups in vivo. (**E**–**H**) Western blot analysis of the expression levels of cleaved caspase-3, cleaved caspase-9, and Bax/Bcl-2 for the in vitro experiment. (**I**,**J**) Hoechst 33258 staining showed that apoptosis of cardiomyocyte was relieved by melatonin treatment. The data are presented as means ± standard deviation (**B**–**D**,**F**–**H**,**J**) and representative blots or images (**A**,**E**,**I**). All experiments were performed in triplicate, at least. * *p* < 0.05, *** *p* < 0.001. NG, normal glucose group (5.5 mM); HG, high glucose group (33 mM). Statistical analyses were performed using one-way ANOVA with Tukey’s multiple comparisons test.

Western blotting was applied to detect some of the proteins associated with apoptosis in diabetic rats and H9c2 cells in high glucose condition. In vivo, the results show that the expressions of cleaved caspase-3, cleaved caspase-9, and Bax/bcl-2 were obviously upregulated in the DM group compared with the normal group, which indicates that hyperglycemia induced severe apoptosis. Surprisingly, the expressions of several proteins were normalizing with melatonin treatment (Figure 2A–D). In vitro, the expressions of cleaved caspase-3, cleaved caspase-9, and BAX/Bcl-2 also increased remarkably in H9c2 cells in high glucose condition. Consistent with the results in vivo, the changes in apoptotic protein expression were reversed via melatonin treatment (Figure 2E–H). We also demonstrate that melatonin alleviated hyperglycemia-induced apoptosis in H9c2 by Hoechst 33258 staining (Figure 2I,J).

### 2.3. Melatonin Suppressed Phosphorylation of JNK/ERK/p38 in Diabetic Cardiomyocytes

**Figure 3:**
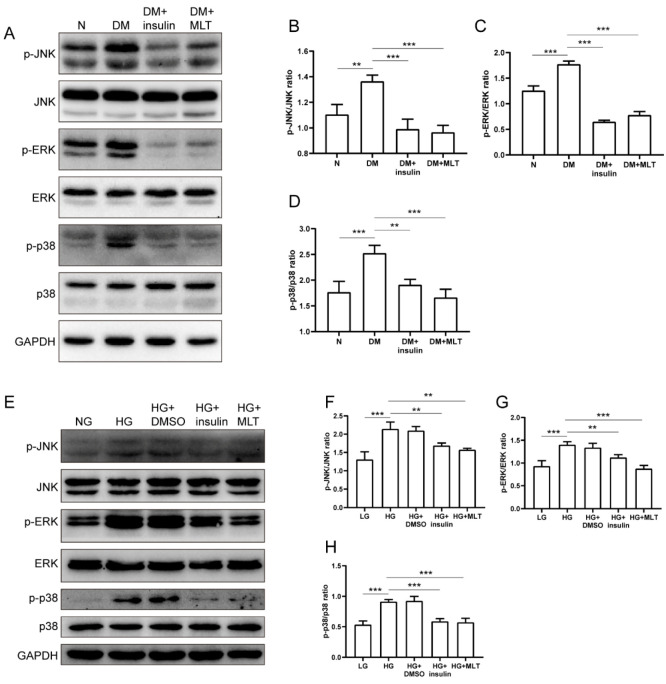
Melatonin reduced phosphorylated levels of JNK/ERK/p38 in diabetic myocardium and H9c2 cells. (**A**–**D**) The phosphorylated levels of JNK/ERK/p38 in myocardium are shown in different groups in vivo. (**E**–**H**) The phosphorylated levels of JNK/ERK/p38 in cardiomyocytes were showed in different groups in vitro. The data are presented as means ± standard deviation (**B**–**D**,**F**–**H**) and representative blots or images (**A**,**E**). All experiments were performed in triplicate at least. ** *p* < 0.01, *** *p* < 0.001. Statistical analyses were performed using one-way ANOVA with Tukey’s multiple comparisons test.

In the DM group, the phosphorylation levels of JNK/ERK/p38 were significantly increased in the myocardium compared with the normal group. Whereas, after insulin and melatonin treatment, the phosphorylation levels of JNK/ERK/p38 were inhibited strikingly (Figure 3A–D). In H9c2 cells, the expressions of p-JNK, p-ERK, and p-p38 were remarkably upregulated with the stimulus of high glucose. Meanwhile, the phosphorylation levels of the above-mentioned proteins decreased dramatically with the administration of insulin and melatonin (Figure 3E–H).

### 2.4. Melatonin Regulated the Complex Network of lncRNA H19, miRNA-29c, and MAPK13 in Cardiomyocytes

**Figure 4:**
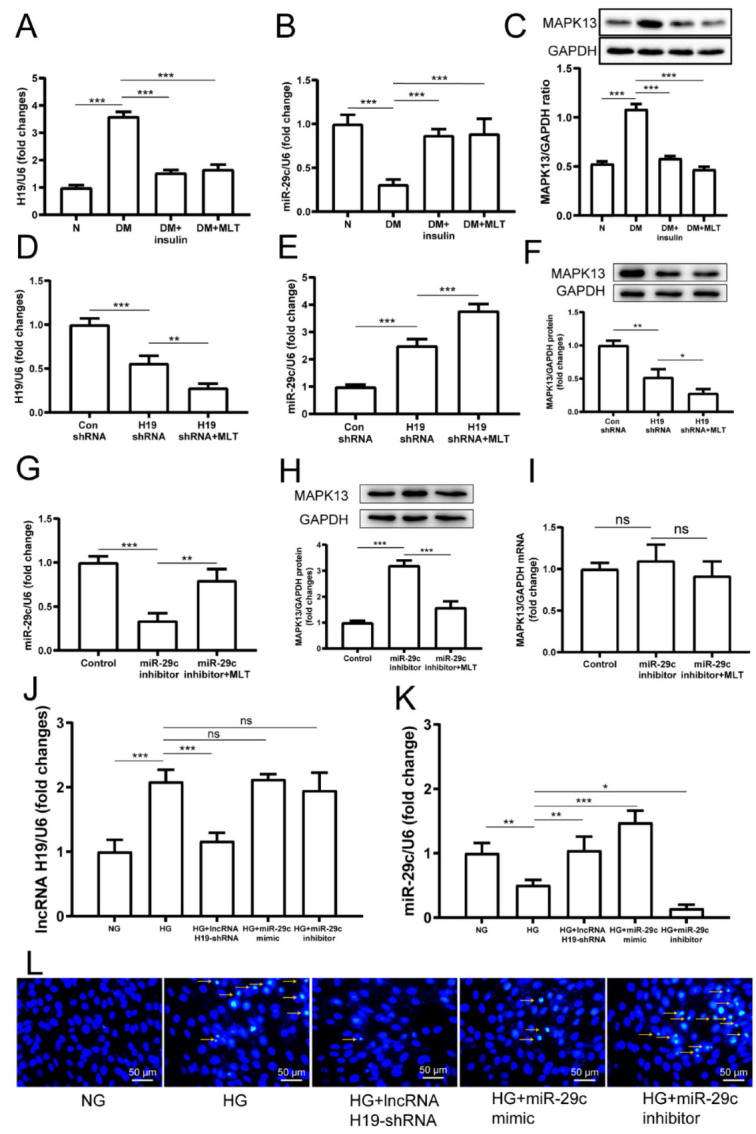
Melatonin affected the expressions of lncRNA H19 and miR-29c in the diabetic myocardium, and regulated the networks of lncRNA H19, miR-29c, and MAPK13 in H9c2 cells. The higher expression of lncRNA H19 (**A**) and MAPK13 protein (**C**), and lower expression of miR-29c (**B**), was abolished by melatonin treatment. The lncRNA H19 (**D**), miR-29c (**E**), and MAPK13 protein (**F**) levels changed significantly with lncRNA H19-shRNA and melatonin treatments. Obvious expression changes for miR-29c (**G**) and MAPK13 protein (**H**) appeared in H9c2 cells with miR-29c inhibitor and melatonin treatments, but there was no change in MAPK13 mRNA expression (**I**). The expression of lncRNA H19 (**J**) and miR-29c (**K**) after lncRNA H19-shRNA, miR-29c mimic, and inhibitor treatment, respectively. The apoptosis of cardiomyocytes (**L**) with lncRNA H19-shRNA, miR-29c mimic, and inhibitor treatment, respectively. The data are presented as means ± standard deviation (**A**–**K**) and representative blots or images (**C**,**F**,**H**,**L**). All experiments were performed in triplicate, at least. * *p* < 0.05, ** *p* < 0.01, *** *p* < 0.001, ns: non-significant. Statistical analyses were performed using one-way ANOVA with Tukey’s multiple comparisons test.

In diabetic rats, the expression of lncRNA H19 in the myocardium was significantly downregulated after treatment with melatonin (Figure 4A). Conversely, the expression of miRNA-29c in the diabetic myocardium was dramatically upregulated by melatonin (Figure 4B). Furthermore, the MAPK13 protein was significantly decreased with melatonin treatment (Figure 4C). In vitro, the expression of lncRNA H19 in H9c2 was significantly inhibited with lncRNA H19-shRNA treatment, and lncRNA H19 expression was further inhibited with both lncRNA H19-shRNA and melatonin treatment (Figure 4D), indicating that melatonin inhibited lncRNA H19 expression. On the contrary, the expression of miR-29c was obviously upregulated with both lncRNA H19-shRNA and melatonin treatments (Figure 4E). Intriguingly, MAPK13 protein expression was also significantly decreased after lncRNA H19 downregulation, and melatonin further suppressed MAPK13 expression in H9c2 (Figure 4F). With the miR-29c inhibitor treatment, the expression of miR-29c was distinctly suppressed, whereas it rose back with melatonin treatment simultaneously (Figure 4G), indicating that melatonin promoted miR-29c expression. In contrast with lncRNA H19-shRNA, the miR-29c inhibitor increased MAPK13 protein expression in H9c2; however, melatonin reversed the effect of the miR-29c inhibitor (Figure 4H). As expected, MAPK13 expression was affected at a post-transcriptional level (Figure 4I). The expression of lncRNA H19 had no significant change with miR-29c mimic or inhibitor treatment (Figure 4J). The expression of miR-29c was upregulated with mimic treatment, while inhibitor treatment downregulated miR-29c expression (Figure 4K). Compared with the normal glucose group, high glucose treatment induced apoptosis in H9c2 cells. Transfection of lncRNA H19-shRNA significantly alleviated apoptosis. Similarly, miR-29c mimic treatment also ameliorated apoptosis in H9c2 cells. In contrast, after miR-29c inhibitor treatment, apoptosis in H9c2 cells increased (Figure 4L). The above results imply that there are potential relationships among lncRNA H19/miR-29c, and MAPK signal pathways, and that melatonin plays a role in the regulation of apoptosis.

### 2.5. LncRNA H19 Binds miR-29c Directly and MAPK13 Is a Target of miR-29c

**Figure 5:**
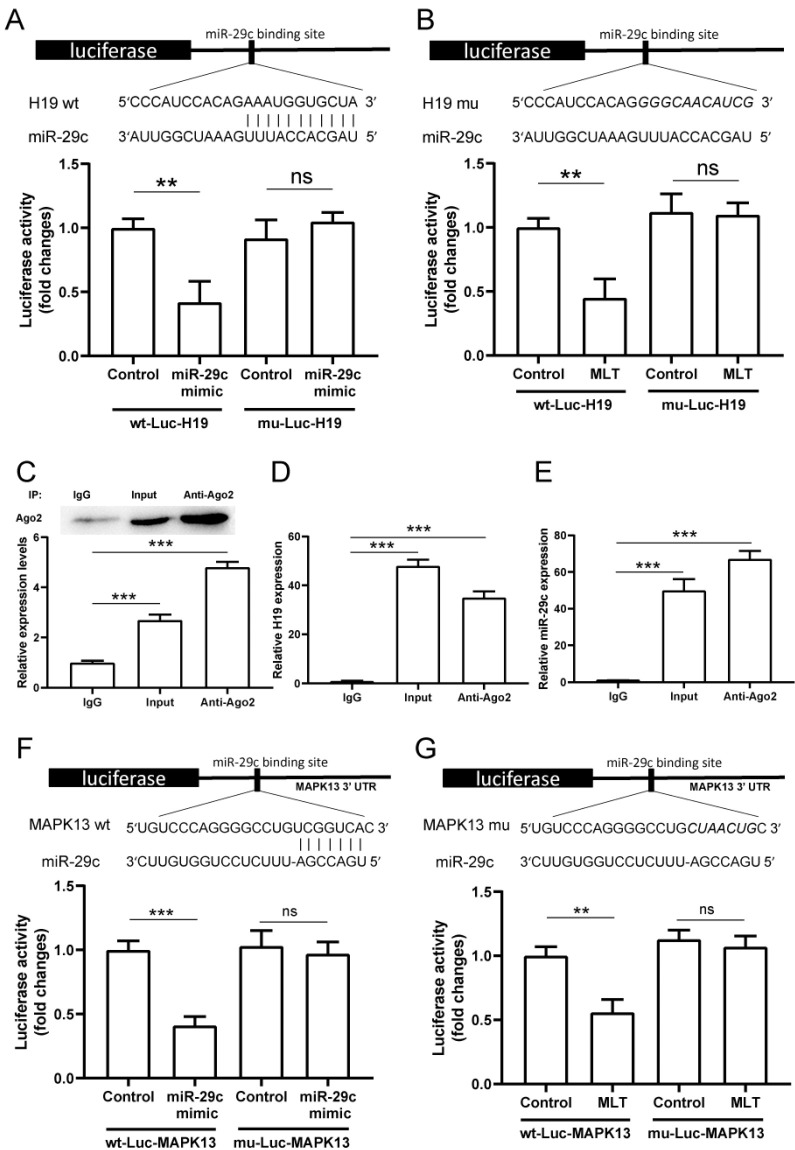
The potential miR-29c binding sites in lncRNA H19 and MAPK13. (**A**) Wildtype lncRNA H19 binds with miR-29c. MiR-29c mimic and luciferase constructs were co-transfected into H9c2 cells. Mutated lncRNA H19 could not target MAPK13 in the presence of the miR-29c mimic. (**B**) Melatonin combined with luciferase constructs were added to H9c2 cells. Cellular lysate from H9c2 was used for RNA immunoprecipitation with Ago2 antibody. Ago2 protein levels were measured by Western blotting (**C**), and the expressions of lncRNA H19 (**D**) and miR-29c (**E**) in the immunoprecipitate were measured by RT-PCR. The miR-29c binding sites in wildtype and mutated MAPK13 3′-UTR are shown (**F**,**G**). MiR-29c mimic and melatonin were separately added to H9c2 cells with luciferase constructs. The data are presented as means ± standard deviation. All experiments were performed in triplicate, at least. ** *p* < 0.01, *** *p* < 0.001, ns: non-significant. Statistical analyses were performed using unpaired *t*-tests (**A**,**B**,**F**,**G**) and one-way ANOVA with Tukey’s multiple comparisons test (**C**,**D**,**E**).

The luciferase assay results indicate that the miR-29c mimic treatment induced a decrease in wildtype lncRNA H19 luciferase activity. However, this effect disappeared when a certain lncRNA H19 site mutated, and there was no significant change between the miR-29c mimic and control groups (Figure 5A). Treatment with melatonin induced an analogous result. Melatonin reduced luciferase activity of wildtype lncRNA H19 rather than mutational lncRNA H19 (Figure 5B). The RNA immunoprecipitation (RIP) experiment was performed to investigate whether lncRNA H19 and miR-29c were components of the RNA-induced silencing complex. Members of the Ago gene family can combine with miRNA to form a complex (RISC), and degrade target mRNA or inhibit translation. For example, Ago2, a miRNA-binding protein, is often used to purify miRNA in RIP experiments, indicating that miRNA can bind to lncRNA. An Ago2 antibody was used to precipitate Ago2 protein from cultured cells (Figure 5C). The mRNA expressions of both lncRNA H19 and miR-29c were significantly high in the immunoprecipitate (Figure 5D,E), suggesting that lncRNA H19 binds miR-29c directly. Meanwhile, results from another luciferase assay show that the miRNA29c mimic caused the inhibition of wildtype MPAK13 luciferase activity. This effect was reduced after mutation at a certain MAPK13 site (Figure 5F). Similarly, the melatonin treatment decreased wildtype MAPK13 rather than mutational MAPK13 luciferase activity (Figure 5G).

### 2.6. LncRNA H19-shRNA, miRNA-29c Mimic, and Melatonin Treatments Ameliorated Hyperglycemia-Induced Apoptosis in H9c2 Cells

**Figure 6:**
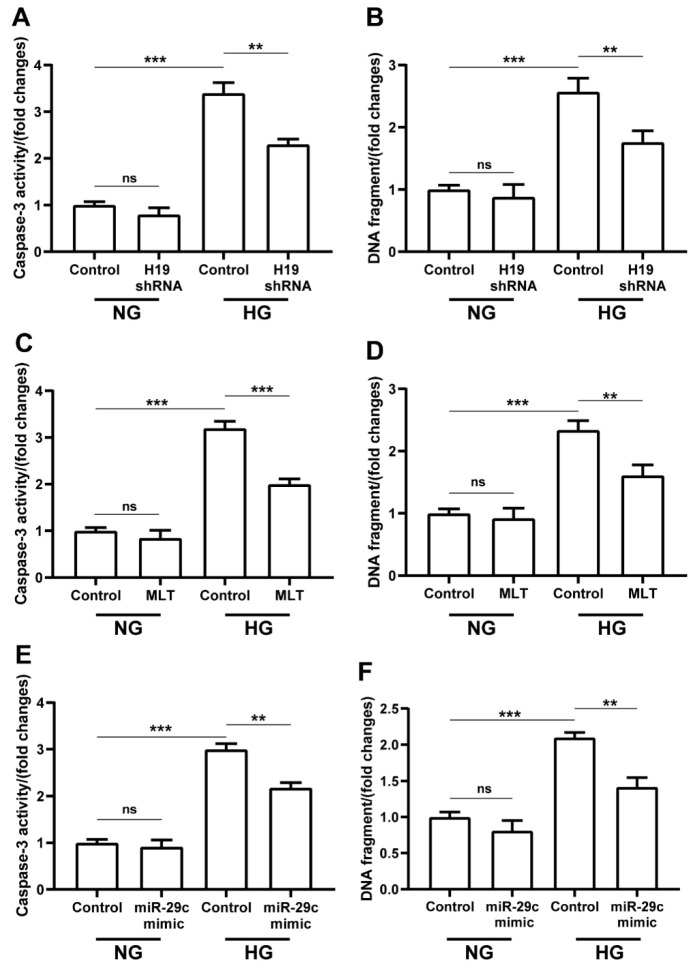
The effects of lncRNA H19 shRNA, melatonin, and miR-29c mimic on apoptosis in H9c2 cells cultured in NG (NG: 5.5 mM) or HG (HG: 33 mM) medium. The decrease in caspase-3 activity (**A**,**C**,**E**) and DNA fragmentation (**B**,**D**,**F**) indicated protective effects of the above treatments against apoptosis of cells in hyperglycemic condition. The data are presented as means ± standard deviation. All experiments were performed in triplicate, at least. ** *p* < 0.01, *** *p* < 0.001, ns: non-significant. Statistical analyses were performed using unpaired *t*-tests.

Caspase-3 activity and DNA fragment assays were performed to determine the effects of lncRNA H19-shRNA, miRNA-29c mimic, and melatonin on cell apoptosis. High glucose condition could cause increased caspase-3 activity and DNA fragmentation in H9c2 cells (Figure 6A). With transfection of lncRNA H19-shRNA, caspase-3 activity was significantly downregulated in H9c2 cells in high glucose condition (Figure 6A). Similar to caspase-3 activity, DNA fragmentation was also considerably downregulated in high-glucose-treated cells with lncRNA H19-shRNA transfection (Figure 6B). Interestingly, we obtained consistent results for melatonin and miRNA-29c mimic treatment, which showed significant decreases in caspase-3 activity and DNA fragmentation (Figure 6C–F).

### 2.7. Melatonin and lncRNA H19-shRNA Reversed the Pro-Apoptotic Effect of miRNA-29c Inhibitor in High-Glucose-Treated H9c2 Cells

**Figure 7:**
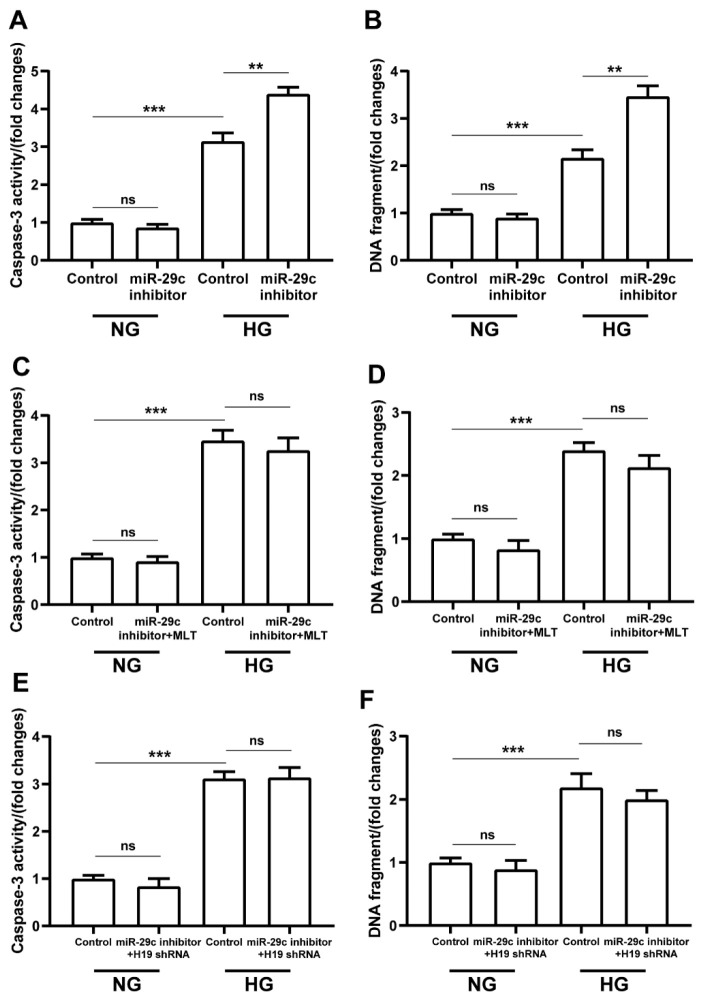
The effects of miR-29c inhibitor alone and in combination with lncRNA H19-shRNA or melatonin on apoptosis of H9c2 cells in hyperglycemic condition. (**A**,**B**) Data show the pro-apoptotic effect of miR-29c inhibitor on H9c2 cells in hyperglycemic condition. (**C**–**F**) Melatonin and lncRNA H19-shRNA reversed the pro-apoptotic effect of miR-29c inhibitor on H9c2 cells in hyperglycemic condition. The data are presented as means ± standard deviation. All experiments were performed in triplicate, at least. ** *p* < 0.01, *** *p* < 0.001, ns: non-significant. Statistical analyses were performed using unpaired *t*-tests.

Compared with the normal glucose group, we detected significant increases in caspase-3 activity and DNA fragmentation in the high glucose group with miR-29c inhibitor transfection alone (Figure 7A,B). We also observed remarkable inhibition of caspase-3 activity and DNA fragmentation with melatonin and lncRNA H19-shRNA treatment, respectively, in high-glucose groups (Figure 7C–F). In brief, the pro-apoptotic effect of miR-29c inhibitor treatment was reversed by melatonin and lncRNA H19-shRNA treatments for cells in hyperglycemic condition.

## 3. Discussion

Currently, there is a lack of effective medical therapies to halt the relentless progression of DCM. Thus, there is a necessary medical need to detect new therapeutic targets for treating DCM. In STZ-induced diabetic models, persistent hyperglycemia caused cardiac damage in rats, and, subsequently, induced the pathology of DCM [31,32]. During the study, we found that melatonin could alleviate hyperglycemia-induced myocardial damage and cardiac hypofunction. Meanwhile, melatonin also ameliorated changes in lncRNA H19 and miRNA-29c caused by chronic hyperglycemia. Thus, we hypothesized that melatonin regulates the pathology of DCM via the lncRNA H19/miRNA-29c axis. In the present study, we verified that melatonin has an alleviative effect on hyperglycemia-induced cardiomyocyte apoptosis in both in vivo and in vitro models. Our data, for the first time, reveal that the lncRNA H19/miR-29c/MAPK axis plays a pivotal role in the anti-apoptotic effect of melatonin in diabetic cardiomyopathy. This novel discovery indicates the possible therapeutic targets, and proposes the potential clinical application of melatonin for DCM treatment. As shown in the graphical abstract, melatonin maintains ncRNAs homeostasis and reduces cardiomyocyte apoptosis of cells in hyperglycemic condition via lncRNA H19/miR-29c/MAPK axis.

The high risks associated with cardiovascular diseases have attracted much attention in diabetic patients [33,34]. Long-term hyperglycemia has been proposed to induce damage and cause subsequent apoptosis in cardiomyocytes [35,36]. It has been reported that cardiomyocyte apoptosis exacerbated the development of DCM [37,38,39]. In our previous research, we found that persistent hyperglycemia aggravated the endoplasmic reticulum stress response of cardiomyocytes, which, in turn, led to cell apoptosis [27]. It was reported that hyperglycemia-induced activation of JNK and p38 MAPK signaling pathways stimulated the expression of apoptosis-related proteins, such as caspase-3, which, in turn, activated the apoptotic pathway and caused apoptosis of cardiomyocytes. However, when the activity of phosphorylation of JNK and p38 MAPK were suppressed, cell apoptosis was also attenuated in diabetic cardiomyopathy models [40,41,42]. In this study, we found that the 3′-UTR of MAPK13 was a target of miR-29c, using bioinformatics analysis. Our previous articles reported that the MAPK signaling pathway was related to cardiovascular diseases such as atherosclerosis; therefore, we chose MAPK13 as the target [43]. Furthermore, we confirmed that MAPK signal pathways in cardiomyocytes were activated under hyperglycemic condition, which may elevate caspase-3 and -9 dependent apoptosis-related proteins expression and promote progression of apoptosis in diabetic myocardium.

Melatonin is known as its crucial role in sleep and circadian rhythms regulation. People increasingly recognized the importance of a well-functioning circadian rhythm system for health maintenance. Additionally, melatonin plays an important role in many areas, such as the immune system, cancer, and mood disorders [44,45]. What is striking is that melatonin has been proposed as a potential therapeutic agent for DCM on account of its multiple physiological functions. Melatonin could exhibit antioxidative stress activity via decreasing mTOR signaling pathway activation, and restore the impaired mitophagy activity by suppressing Mst1 in animals with DCM [46,47]. It has also been published that melatonin could protect the rat heart against diabetes-induced apoptosis by ameliorating metabolic risk factors and modulating apoptotic proteins [48]. Our former research also revealed that melatonin plays a positive role against apoptosis via regulating endoplasmic reticulum stress and MAPK pathways [27,28]. In this study, our data show that melatonin could not directly decrease blood glucose levels in diabetic rats, but it could distinctly ameliorate diabetic cardiac dysfunction independent of the regulation of blood glucose level, perhaps partly through reducing myocardial apoptosis via the modulation of MAPK signal pathways and apoptosis-related proteins. We found that melatonin could improve cardiac dysfunction caused by hyperglycemia using the Langendorff perfusion system. Consistent with the parameters of cardiac function, the results of morphological and staining analyses further confirmed that diabetes-induced myocardial tissue damage can be cured with melatonin treatment. However, the underlying molecular mechanisms of melatonin in hyperglycemia-induced cardiomyocyte apoptosis are yet to be elucidated.

The competing endogenous RNA (ceRNA) networks, including lncRNAs and miRNAs interactions, were reportedly involved in the regulation of protein-coding genes implicated in the pathological process of DCM [14,49,50,51,52]. LncRNA H19, one of the lncRNAs abundant in the cardiovascular system, was reported to act as a ceRNA on different miRNAs to exert effects on cardiovascular complications [53,54]. Our previous study showed that lncRNA H19 suppression protected the endothelium against hyperglycemia-induced inflammation and oxidative stress by upregulating miR-29b expression and downregulating VEGFA expression, which caused the activation of the AKT/eNOS pathway in endothelial cells [11]. In this study, our results show that lncRNA H19 expression was negatively associated with miR-29c expression in myocardia of diabetic rats. Although it has been widely reported that melatonin alleviates diabetic cardiomyopathy at the protein level, the effects of melatonin on ceRNAs have rarely been covered. Importantly, our study confirms that melatonin partly inverses the increased lncRNA H19 and decreases miR-29c levels in diabetic cardiomyocytes. Moreover, our vitro experiments indicate that lncRNA H19 binds to miR-29c as a sponge, and they interact directly. The silencing of lncRNA H19 further increased miR-29c expression in cardiomyocytes. We also observed that silencing of lncRNA H19 suppressed the expression of MAPK13 protein in cardiomyocytes, which was testified in several studies. For example, lncRNA H19 can target certain RNAs to regulate MAPK signal pathways and promote the development of atherosclerosis and arterial calcification [16,55]. On the contrary, our data show that the inhibition of miR-29c expression stimulated MAPK13 expression in cardiomyocytes, and the luciferase assay results demonstrate that MAPK13 genes were the direct target of miR-29c. Thus, the above results reveal that lncRNA H19 may, in part, regulate MAPK13 expression by competing with miR-29c. It is noteworthy that melatonin inhibits MAPK13 expression in cardiomyocytes via the modulation of lncRNA19/miR-29c levels, as observed in our study. To investigate the role of the lncRNA H19/miR-29c/MAPK13 signal pathway in the regulation of cardiomyocyte apoptosis, lncRNA H19 shRNA and an miR-29c mimic were transfected into cardiomyocytes in hyperglycemic condition, which led to the alleviation of apoptosis. In light of the role of MAPK signal pathways in apoptosis, we prove the potential involvement of lncRNA H19/miR-29c/MAPK13 signal pathway in the process of cardiomyocyte apoptosis in diabetes. Most importantly, our data show that melatonin ameliorates cardiomyocyte apoptosis through the modulation of the lncRNA H19-mediated ceRNA network, which we confirm for the first time.

However, our experiments have some limitations. For example, in the pathology of diabetic patients, the blood glucose levels rarely reached 33 mM, whereas, during the experiment, the blood glucose levels of diabetic rats were generally higher than 33 mM; thus, we treated H9c2 cells with high glucose (33 mM) to simulate the state of diabetes. For proper controls in the animal experiment, a melatonin treatment group in normoglycemic animals would be needed. Furthermore, it would be more suitable using primary cardiomyocytes or human cardiomyocytes to build vitro models rather than rat H9c2 cardiomyocytes in experiments. In our future research, we intend to add a drug treatment group, using normoglycemic animals as controls. We will also use primary cells as much as possible to complete the in vitro experiments.

Insulin has been widely used to treat diabetes and its complications, and it can regulate DCM through multiple pathways, including its involvement in regulating excessive production of advanced glycation end-products; activating the hexosamine biosynthetic pathway; ameliorating the effects of lipotoxicity, mitochondrial dysfunction, and increased oxidative stress; activating the renin–angiotensin system; and restoring impaired calcium homeostasis [56]. Therefore, insulin was chosen as a positive control, and melatonin was investigated as an adjunctive drug, in the present study. Melatonin can alleviate cardiovascular diseases such as atherosclerosis by regulating oxidative stress, endoplasmic reticulum stress, and the MAPK signaling pathway, et al. In the present study, we found that melatonin alleviated cardiomyocyte apoptosis in DCM via the lncRNA H19/miRNA-29c/MAPK axis. We suggest that the advantage of melatonin is that it will not cause hypoglycemia, and that supplementary therapy with the use of hypoglycemic drugs such as insulin can further alleviate DCM by ameliorating oxidative stress, endoplasmic reticulum stress, and regulating MAPK signaling pathways, et al. We will further explore its advantages in future research.

## 4. Materials and Methods

### 4.1. Animals

All animal experiments were conducted following the Guide for the Use and Care of Laboratory Animals. All experimental protocols were reviewed and approved by the Ethics Committee of Anhui Medical University (Hefei, China). The bioethical commitment approval code is LISC20210825. We built STZ-induced diabetes models in Sprague Dawley (SD) rats. Male SD rats (220–250 g, 8 weeks old) were purchased from Beijing Vital River Laboratory Animal Technology Co., Ltd. (Beijing, China). The rats were acclimatized to the standard laboratory conditions for 1 week, with normal water and chow available ad libitum after arrival. Afterwards, rats were randomized into normal, DM, DM + insulin, and DM + MLT groups (12 rats in per group), and they received a single injection of STZ (55 mg/kg, Sigma-Aldrich, St. Louis, MO, USA; Merck KGaA, Darmstadt, Germany), except the normal group. Animals were considered to be diabetic if the blood glucose levels tested higher than 11.1 mmol/L after 1 week. Diabetes was induced in rats by injecting insulin (1 u/kg/d within first 10 weeks once a day, and twice a day for the next 6 weeks, Wanbang Biopharmaceuticals, Xuzhou, China) and melatonin gavage (10 mg/kg/d, Nanjing Duly Biotech Co., Ltd.; Nanjing, China) separately, in DM + insulin and DM + MLT groups. During the experiment, all rats could consume water and chow at liberty. At the end of week 16, the rats were anesthetized with an intraperitoneal injection of ketamine–xylazine solution (80 mg/kg and 10 mg/kg, respectively) and euthanized. With the heart obtained, cardiac function was immediately evaluated by the way of the Langendorff perfusion system; we then collected the myocardial tissues.

### 4.2. Langendorff Perfusion System

The Langendorff perfusion system was used to record cardiac function in rats. Briefly, the heart was rapidly excised by thoracotomy under anesthesia. Then we drained the blood in pre-cooled modified Krebs–Henseleit buffer, and the aorta was cannulated. The isolated heart was mounted onto the Langendorff perfusion system (Chengdu Techman Software Co., Ltd., Chengdu, China) and perfused with modified Krebs-Henseleit buffer aerated with 95% O_2_ and 5% CO_2_, yielding a final pH of 7.4 in a 37 °C circulating water bath. When stable, left ventricular development pressure (LVDP), coronary flow, maximal rate of the increase in left ventricular pressure (+dp/dt max) and maximal rate of the decrease in left ventricular pressure (−dp/dt max), which are related to cardiac function, were recorded via BL-420S biological function experiment system (Chengdu Techman Software Co., Ltd., Chengdu, China).

### 4.3. Histology

The paraffin-embedded myocardial tissues were cut into 5-μm thick sections and stained with H&E (hematoxylin and eosin staining kit, Beyotime, Shanghai, China) or Masson’s trichrome staining kit (Nanjing Jiancheng Technology Co., Ltd., Nanjing, China). The discrepant areas were captured by a DMI4000B fluorescence microscope (Leica, Munich, Germany).

### 4.4. Cell Culture

The cell line H9c2 was obtained from the Cell Bank of Shanghai Institutes for Biological Sciences, Chinese Academy of Sciences. Cells were cultured in low-glucose Dulbecco’s Modified Eagles Media (DMEM) (hyclone, Logan, UT, USA) supplemented with 10% fetal bovine serum (FBS) (gbico, Melbourne, Australia), and 100 IU/mL penicillin/streptomycin (Beyotime, Shanghai, China) in 5% CO_2_ at a temperature of 37 °C. The insulin (1 μM) and melatonin (10 μM) were treated 48 h with high glucose concentration (33 mM).

### 4.5. Cell Transfection

LncRNA H19-shRNA (GenePharm Co., Ltd., Shanghai, China) was used to silence lncRNA H19 expression. Simultaneously, miR-29c mimics and inhibitors (GenePharm) were used to regulate miR-29c expression. H9c2 cells were transfected with lncRNA H19-shRNA, or miR-29c mimics, inhibitors, or controls (GenePharm) with Lipofectamine 2000 (Invitrogen; Thermo Fisher Scientific, Inc., Waltham, MA, USA) according to the manufacturer’s protocol. A scrambled oligonucleotide (GenePharm) served as a control. Changes in RNA expression were determined by RT-qPCR 24 h after transfection, and differences in protein expression were measured via Western blotting 48 h after transfection.

### 4.6. Western Blot Analysis

The tissues and cells were collected in RIPA buffer containing protease and phosphatase inhibitors. Total protein concentration was quantified with BCA Protein Assay Reagent Kit (Beyotime, Shanghai, China). The proteins were separated by SDS–PAGE and transferred onto polyvinylidene difluoride (PVDF) membranes (Millipore, Burlington, MA, USA) via electroblotting. The membranes were blocked in 5% skimmed milk in TBST (TBS, 0.1% Tween 20) for 2 h and incubated overnight at 4 °C with primary antibodies against p- JNK (Santa Cruz Biotechnology, Dallas, TX, USA, Cat# sc-6254), JNK (Santa, Cat# sc-7345), p-ERK (Santa, Cat# sc-7383), ERK (Santa, Cat# sc-135900), p-p38 (Santa, Cat# sc-7975-R), p38 (Santa, Cat# sc-7149), BAX (Santa, Cat# sc-20067), Bcl-2 (Santa, Cat# sc-7382), caspase-3 (Proteintech, Wuhan, China, Cat# 11648-2-AP), caspase-9 (Cell Signaling Technology, Danvers, MA, USA, Cat# 9508S) and GAPDH (Santa, Cat# sc-32233). The PVDF membranes containing proteins were incubated with horseradish peroxidase-conjugated secondary antibodies. Subsequently, target proteins were stained with ECL chemiluminescence detection kit (FDbio Science Biotech Co., Ltd., Hangzhou, China). Densitometric analysis was performed using an imaging station.

### 4.7. Hoechst 33258 Staining

Cells were seeded in 24-well plates, cultured, and treated; the medium was discarded. After washing with PBS three times, cells were fixed in fixative solution for 10 min. The samples were incubated with Hoechst 33258 (Beyotime, Shanghai, China) staining solution for 5 min in darkness. Then, the plates were kept in the dark, observed, and imaged using a fluorescence microscope.

### 4.8. Luciferase Assay

The regions of lncRNA H19 and MAPK13 3′-UTR, including potential miR-29c binding sites, were predicted with TargetScan version 7.11, and amplified by PCR. Subsequently, mutants were constructed by introducing point mutations into the seed binding site for miR-29c. The wildtype and mutant fragments were subcloned into the firefly luciferase-expressing vector. H9c2 cells were seeded in 24-well plates and co-transfected with wildtype or mutated luciferase, respectively. The Dual Luciferase Reporter Assay System (Promega, Madison, WI, USA) was used 48 h after transfection following the manufacturer’s protocol. The relative luciferase activity was calculated according to the ratio of firefly luciferase activity to Renilla luciferase activity.

### 4.9. RNA Immunoprecipitation (RIP)

We investigated the direct interaction between miR-29c and lncRNA H19 by Argonaute 2 (Ago2)-RNA immunoprecipitation (Ago2-RIP). Anti-Ago2 (Sigma-Aldrich, St. Louis, MA, USA), or control anti-IgG and Dynabeads Protein G (Invitrogen, Waltham, MA, USA) were pretreated at 4 °C with rotation 24 h in advance. Complete RIP lysis buffer, which contained protease inhibitor, phosphatase inhibitor and RNase inhibitor, was used to lyse cells. RNA in Ago2-RIP materials was washed several times with PEB buffer and treated with DNase I and Proteinase K. RNA was extracted with TRIzol (Invitrogen) and precipitated with absolute ethanol overnight at −20 °C. After the removal of proteins and beads, RT-qPCR analysis of the purified RNA and lncRNA H19 enrichment in Ago2-RIP was conducted.

### 4.10. Reverse Transcription-Quantitative Polymerase Chain Reaction (RT-qPCR)

RNA from myocardial tissues and H9c2 cells was isolated using TRIzol reagent and converted to complementary DNA (cDNA) using a First-Strand cDNA Synthesis Kit (Toyobo, Osaka, Japan). Then, the cDNA samples, added with Power SYBR green master mix (Applied Biosystems, Foster City, CA, USA), were subjected to RT-qPCR using a StepOne Real Time PCR system. The lncRNA H19, miR-29c and mRNA level were respectively standardized by U6 and GAPDH. The amplification results were calculated on the basis of 2 (−ΔΔCt) method. The specific primers used were: lncRNA H19 forward 5′-ATCGGTGCCTCAGCGTTCGG-3′ and reverse 5′-CTGTCCTCGCCGTCACACCG-3′; MAPK13 forward 5′-GAGAAGGTGGCCATCAAGAA-3′ and reverse 5′-GTCCTCATTCACAGCCAGGT-3; GAPDH forward 5′-GGTGGTCTCCTCTGACTTCAA-3′ and reverse 5′-GTTGCTGTAGCCAAATTCGTTGT-3′; miR-29c, 5′-UAGCACCAUUUGAAAUCAGUGUU-3′; U6, 5′-CGCTTCGGCAGCACATATACTAAAATTGGAAC-3′.

### 4.11. Caspase-3 Activity Assay

Caspase-3 activity was detected using a caspase-3 activity assay kit (Biomol Research Laboratories, Plymouth Meeting, PA, USA) according to the manufacturer’s protocol. After the cells lysed, total proteins were extracted and quantified using a protein assay kit. Subsequently, the proteins were incubated overnight at 37 °C with acetyl-Asp-Glu-Val-Asp p-nitroanilide (Ac-DEVD-pNA) for the caspase-3 assay. The absorbance of pNA was detected by a microplate reader at 405 nm.

### 4.12. DNA Fragment Assay

DNA fragments were measured using a Cellular DNA Fragmentation ELISA kit (Roche Applied Science, Greenfield, IN, USA). Cells were seeded in 96-well plates. The medium was changed to serum-free medium after 24 h, and continued to culture cells for an additional 24 h. To label the DNA, the medium was replaced with 10% FBS-DMEM supplemented with 5-bromo-2′-deoxyuridine. Following 24 h incubated, cells were treated with calcitriol for 4 h and MIS for 96 h. Cells were lysed, and then soluble DNA fragments were quantified by the Cellular DNA fragmentation ELISA kit according to the manufacturer’s instructions.

### 4.13. Statistical Analysis

Statistical analysis was performed using SPSS 20.0. The data were subjected to one-way ANOVA with Tukey’s multiple comparisons test and unpaired parametric *t*-tests, and presented as means ± standard deviation. All experiments were performed at least in triplicate. The results are considered statistically significant when *p* < 0.05.

## 5. Conclusions

In summary, we discovered that melatonin treatment effectively ameliorated cardiac dysfunction and cardiomyocyte apoptosis in DCM, and this protective activity appeared to be largely dependent on the modulation of the lncRNA H19/miR-29c/MAPK axis. These findings provide new insights into the role of melatonin as an attractive agent for alleviating the progression of DCM.

## Data Availability

Data is contained within the article.

## References

[1] Cole J.B., Florez J.C. (2020). Genetics of diabetes mellitus and diabetes complications. Nat. Rev. Nephrol..

[2] Tan Y., Zhang Z., Zheng C., Wintergerst K.A., Keller B.B., Cai L. (2020). Mechanisms of diabetic cardiomyopathy and potential therapeutic strategies: Preclinical and clinical evidence. Nat. Rev. Cardiol..

[3] Murtaza G., Virk H., Khalid M., Lavie C.J., Ventura H., Mukherjee D., Ramu V., Bhogal S., Kumar G., Shanmugasundaram M. (2019). Diabetic cardiomyopathy—A comprehensive updated review. Prog. Cardiovasc. Dis..

[4] Seferović P.M., Paulus W.J. (2015). Clinical diabetic cardiomyopathy: A two-faced disease with restrictive and dilated phenotypes. Eur. Heart J..

[5] Shah S.J., Kitzman D.W., Borlaug B.A., van Heerebeek L., Zile M.R., Kass D.A., Paulus W.J. (2016). Phenotype-Specific Treatment of Heart Failure with Preserved Ejection Fraction: A Multiorgan Roadmap. Circulation.

[6] Yao Q., Ke Z.Q., Guo S., Yang X.S., Zhang F.X., Liu X.F., Chen X., Chen H.G., Ke H.Y., Liu C. (2018). Curcumin protects against diabetic cardiomyopathy by promoting autophagy and alleviating apoptosis. J. Mol. Cell. Cardiol..

[7] Wang X., Pan J., Liu D., Zhang M., Li X., Tian J., Liu M., Jin T., An F. (2019). Nicorandil alleviates apoptosis in diabetic cardiomyopathy through PI3K/Akt pathway. J. Cell Mol. Med..

[8] Zhang M., Lin J., Wang S., Cheng Z., Hu J., Wang T., Man W., Yin T., Guo W., Gao E. (2017). Melatonin protects against diabetic cardiomyopathy through Mst1/Sirt3 signaling. J. Pineal Res..

[9] Malek V., Gaikwad A.B. (2019). Telmisartan and thiorphan combination treatment attenuates fibrosis and apoptosis in preventing diabetic cardiomyopathy. Cardiovasc. Res..

[10] Yin Z., Zhao Y., He M., Li H., Fan J., Nie X., Yan M., Chen C., Wang D.W. (2019). MiR-30c/PGC-1β protects against diabetic cardiomyopathy via PPARα. Cardiovasc. Diabetol..

[11] Cheng X.W., Chen Z.F., Wan Y.F., Zhou Q., Wang H., Zhu H.Q. (2019). Long Non-coding RNA H19 Suppression Protects the Endothelium Against Hyperglycemic-Induced Inflammation via Inhibiting Expression of miR-29b Target Gene Vascular Endothelial Growth Factor a through Activation of the Protein Kinase B/Endothelial Nitric Oxide Synthase Pathway. Front. Cell Dev. Biol..

[12] Yang S., Tang W., He Y., Wen L., Sun B., Li S. (2018). Long non-coding RNA and microRNA-675/let-7a mediates the protective effect of melatonin against early brain injury after subarachnoid hemorrhage via targeting TP53 and neural growth factor. Cell Death Dis..

[13] Rupaimoole R., Slack F.J. (2017). MicroRNA therapeutics: Towards a new era for the management of cancer and other diseases. Nat. Rev. Drug Discov..

[14] Zhou X., Zhang W., Jin M., Chen J., Xu W., Kong X. (2017). lncRNA MIAT functions as a competing endogenous RNA to upregulate DAPK2 by sponging miR-22-3p in diabetic cardiomyopathy. Cell Death Dis..

[15] Wang C., Liu G., Yang H., Guo S., Wang H., Dong Z., Li X., Bai Y., Cheng Y. (2021). MALAT1-mediated recruitment of the histone methyltransferase EZH2 to the microRNA-22 promoter leads to cardiomyocyte apoptosis in diabetic cardiomyopathy. Sci. Total Environ..

[16] Pan J.X. (2017). LncRNA H19 promotes atherosclerosis by regulating MAPK and NF-kB signaling pathway. Eur. Rev. Med. Pharmacol. Sci..

[17] Bitarafan S., Yari M., Broumand M.A., Ghaderian S., Rahimi M., Mirfakhraie R., Azizi F., Omrani M.D. (2019). Association of Increased Levels of lncRNA H19 in PBMCs with Risk of Coronary Artery Disease. Cell J..

[18] Li T., Gu J., Yang O., Wang J., Wang Y., Kong J. (2020). Bone Marrow Mesenchymal Stem Cell-Derived Exosomal miRNA-29c Decreases Cardiac Ischemia/Reperfusion Injury Through Inhibition of Excessive Autophagy via the PTEN/Akt/mTOR Signaling Pathway. Circ. J..

[19] Hu Y., Deng F., Song J., Lin J., Li X., Tang Y., Zhou J., Tang T., Zheng L. (2015). Evaluation of miR-29c inhibits endotheliocyte migration and angiogenesis of human endothelial cells by suppressing the insulin like growth factor 1. Am. J. Transl. Res..

[20] Reiter R.J., Mayo J.C., Tan D.X., Sainz R.M., Alatorre-Jimenez M., Qin L. (2016). Melatonin as an antioxidant: Under promises but over delivers. J. Pineal Res..

[21] Gonzaga N.A., Awata W., Ficher S.P., Assis V.O., Alves J.V., Tostes R.C., Tirapelli C.R. (2021). Melatonin reverses the loss of the anticontractile effect of perivascular adipose tissue in obese rats. J. Pineal Res..

[22] Tan D.X., Reiter R.J. (2020). An evolutionary view of melatonin synthesis and metabolism related to its biological functions in plants. J. Exp. Bot..

[23] Jilg A., Bechstein P., Saade A., Dick M., Li T.X., Tosini G., Rami A., Zemmar A., Stehle J.H. (2019). Melatonin modulates daytime-dependent synaptic plasticity and learning efficiency. J. Pineal Res..

[24] Yin J., Li Y., Han H., Ma J., Liu G., Wu X., Huang X., Fang R., Baba K., Bin P. (2020). Administration of Exogenous Melatonin Improves the Diurnal Rhythms of the Gut Microbiota in Mice Fed a High-Fat Diet. mSystems.

[25] Yu L.M., Dong X., Xue X.D., Xu S., Zhang X., Xu Y.L., Wang Z.S., Wang Y., Gao H., Liang Y.X. (2021). Melatonin attenuates diabetic cardiomyopathy and reduces myocardial vulnerability to ischemia-reperfusion injury by improving mitochondrial quality control: Role of SIRT6. J. Pineal Res..

[26] Ding M., Feng N., Tang D., Feng J., Li Z., Jia M., Liu Z., Gu X., Wang Y., Fu F. (2018). Melatonin prevents Drp1-mediated mitochondrial fission in diabetic hearts through SIRT1-PGC1α pathway. J. Pineal Res..

[27] Xiong F.Y., Tang S.T., Su H., Tang H.Q., Jiang P., Zhou Q., Wang Y., Zhu H.Q. (2018). Melatonin ameliorates myocardial apoptosis by suppressing endoplasmic reticulum stress in rats with long-term diabetic cardiomyopathy. Mol. Med. Rep..

[28] Tang S.T., Su H., Zhang Q., Tang H.Q., Wang C.J., Zhou Q., Wei W., Zhu H.Q., Wang Y. (2016). Melatonin Attenuates Aortic Endothelial Permeability and Arteriosclerosis in Streptozotocin-Induced Diabetic Rats: Possible Role of MLCK- and MLCP-Dependent MLC Phosphorylation. J. Cardiovasc. Pharmacol. Ther..

[29] Lei Y., Xu Q., Zeng B., Zhang W., Zhen Y., Zhai Y., Cheng F., Mei W., Zheng D., Feng J. (2017). Angiotensin-(1-7) protects cardiomyocytes against high glucose-induced injuries through inhibiting reactive oxygen species-activated leptin-p38 mitogen-activated protein kinase/extracellular signal-regulated protein kinase 1/2 pathways, but not the leptin-c-Jun N-terminal kinase pathway in vitro. J. Diabetes Investig..

[30] Zhao H.L., Wu B.Q., Luo Y., Zhang W.Y., Hao Y.L., Liang J.J., Fang F., Liu W., Chen X.H. (2018). Exogenous hydrogen sulfide ameliorates high glucose-induced myocardial injury & inflammation via the CIRP-MAPK signaling pathway in H9c2 cardiac cells. Life Sci..

[31] Ares-Carrasco S., Picatoste B., Camafeita E., Carrasco-Navarro S., Zubiri I., Ortiz A., Egido J., López J.A., Tuñón J., Lorenzo O. (2012). Proteome changes in the myocardium of experimental chronic diabetes and hypertension: Role of PPARα in the associated hypertrophy. J. Proteom..

[32] Ward M.L., Crossman D.J. (2014). Mechanisms underlying the impaired contractility of diabetic cardiomyopathy. World J. Cardiol..

[33] Cai X., Zhang Y., Li M., Wu J.H., Mai L., Li J., Yang Y., Hu Y., Huang Y. (2020). Association between prediabetes and risk of all cause mortality and cardiovascular disease: Updated meta-analysis. BMJ.

[34] Kenny H.C., Abel E.D. (2019). Heart Failure in Type 2 Diabetes Mellitus. Circ. Res..

[35] Ren B.C., Zhang Y.F., Liu S.S., Cheng X.J., Yang X., Cui X.G., Zhao X.R., Zhao H., Hao M.F., Li M.D. (2020). Curcumin alleviates oxidative stress and inhibits apoptosis in diabetic cardiomyopathy via Sirt1-Foxo1 and PI3K-Akt signalling pathways. J. Cell. Mol. Med..

[36] Xu D., Zhang X., Chen X., Yang S., Chen H. (2020). Inhibition of miR-223 attenuates the NLRP3 inflammasome activation, fibrosis, and apoptosis in diabetic cardiomyopathy. Life Sci..

[37] Qi B., He L., Zhao Y., Zhang L., He Y., Li J., Li C., Zhang B., Huang Q., Xing J. (2020). Akap1 deficiency exacerbates diabetic cardiomyopathy in mice by NDUFS1-mediated mitochondrial dysfunction and apoptosis. Diabetologia.

[38] Chen H., Tran D., Yang H.C., Nylander S., Birnbaum Y., Ye Y. (2020). Dapagliflozin and Ticagrelor Have Additive Effects on the Attenuation of the Activation of the NLRP3 Inflammasome and the Progression of Diabetic Cardiomyopathy: An AMPK-mTOR Interplay. Cardiovasc. Drugs Ther..

[39] Ge Q., Zhao L., Ren X.M., Ye P., Hu Z.Y. (2019). LCZ696, an angiotensin receptor-neprilysin inhibitor, ameliorates diabetic cardiomyopathy by inhibiting inflammation, oxidative stress and apoptosis. Exp. Biol. Med..

[40] Al-Damry N.T., Attia H.A., Al-Rasheed N.M., Al-Rasheed N.M., Mohamad R.A., Al-Amin M.A., Dizmiri N., Atteya M. (2018). Sitagliptin attenuates myocardial apoptosis via activating LKB-1/AMPK/Akt pathway and suppressing the activity of GSK-3β and p38α/MAPK in a rat model of diabetic cardiomyopathy. Biomed. Pharmacother..

[41] Zuo G., Ren X., Qian X., Ye P., Luo J., Gao X., Zhang J., Chen S. (2019). Inhibition of JNK and p38 MAPK-mediated inflammation and apoptosis by ivabradine improves cardiac function in streptozotocin-induced diabetic cardiomyopathy. J. Cell. Physiol..

[42] Xiong Z., Li Y., Zhao Z., Zhang Y., Man W., Lin J., Dong Y., Liu L., Wang B., Wang H. (2020). Mst1 knockdown alleviates cardiac lipotoxicity and inhibits the development of diabetic cardiomyopathy in db/db mice. Biochim. Biophys. Acta (BBA) Mol. Basis Dis..

[43] Lu Z., Wang F., Yu P., Wang X., Wang Y., Tang S.T., Zhu H.Q. (2018). Inhibition of miR-29b suppresses MAPK signaling pathway through targeting SPRY1 in atherosclerosis. Vasc. Pharmacol..

[44] Hardeland R. (2017). Melatonin and the pathologies of weakened or dysregulated circadian oscillators. J. Pineal Res..

[45] McHill A.W., Sano A., Hilditch C.J., Barger L.K., Czeisler C.A., Picard R., Klerman E.B. (2021). Robust stability of melatonin circadian phase, sleep metrics, and chronotype across months in young adults living in real-world settings. J. Pineal Res..

[46] Kandemir Y.B., Tosun V., Güntekin Ü. (2019). Melatonin protects against streptozotocin-induced diabetic cardiomyopathy through the mammalian target of rapamycin (mTOR) signaling pathway. Adv. Clin. Exp. Med..

[47] Wang S., Zhao Z., Feng X., Cheng Z., Xiong Z., Wang T., Lin J., Zhang M., Hu J., Fan Y. (2018). Melatonin activates Parkin translocation and rescues the impaired mitophagy activity of diabetic cardiomyopathy through Mst1 inhibition. J. Cell. Mol. Med..

[48] Amin A.H., El-Missiry M.A., Othman A.I. (2015). Melatonin ameliorates metabolic risk factors, modulates apoptotic proteins, and protects the rat heart against diabetes-induced apoptosis. Eur. J. Pharmacol..

[49] Che H., Wang Y., Li H., Li Y., Sahil A., Lv J., Liu Y., Yang Z., Dong R., Xue H. (2020). Melatonin alleviates cardiac fibrosis via inhibiting lncRNA MALAT1/miR-141-mediated NLRP3 inflammasome and TGF-β1/Smads signaling in diabetic cardiomyopathy. FASEB J..

[50] Sun H., Wang C., Zhou Y., Cheng X. (2020). Long noncoding RNA OIP5-AS1 overexpression promotes viability and inhibits high glucose-induced oxidative stress of cardiomyocytes by targeting microRNA-34a/SIRT1 axis in diabetic cardiomyopathy. Endocr. Metab. Immune Disord. Drug Targets.

[51] Feng Y., Xu W., Zhang W., Wang W., Liu T., Zhou X. (2019). LncRNA DCRF regulates cardiomyocyte autophagy by targeting miR-551b-5p in diabetic cardiomyopathy. Theranostics.

[52] Yang F., Qin Y., Lv J., Wang Y., Che H., Chen X., Jiang Y., Li A., Sun X., Yue E. (2018). Silencing long non-coding RNA Kcnq1ot1 alleviates pyroptosis and fibrosis in diabetic cardiomyopathy. Cell Death Dis..

[53] Shi S., Song L., Yu H., Feng S., He J., Liu Y., He Y. (2020). Knockdown of LncRNA-H19 Ameliorates Kidney Fibrosis in Diabetic Mice by Suppressing miR-29a-Mediated EndMT. Front. Pharmacol..

[54] Fawzy M.S., Abdelghany A.A., Toraih E.A., Mohamed A.M. (2020). Circulating long noncoding RNAs H19 and GAS5 are associated with type 2 diabetes but not with diabetic retinopathy: A preliminary study. Bosn. J. Basic Med. Sci..

[55] Liu F., Yang X.C., Chen M.L., Zhuang Z.W., Jiang Y., Wang J., Zhou Y.J. (2020). LncRNA H19/Runx2 axis promotes VSMCs transition via MAPK pathway. Am. J. Transl Res..

[56] Zamora M., Villena J.A. (2019). Contribution of Impaired Insulin Signaling to the Pathogenesis of Diabetic Cardiomyopathy. Int. J. Mol. Sci..

